# Live *Faecalibacterium prausnitzii* Does Not Enhance Epithelial Barrier Integrity in an Apical Anaerobic Co-Culture Model of the Large Intestine

**DOI:** 10.3390/nu9121349

**Published:** 2017-12-12

**Authors:** Eva Maier, Rachel C. Anderson, Nicole C. Roy

**Affiliations:** 1 Food Nutrition & Health Team, Food & Bio-Based Products Group, AgResearch Grasslands, Palmerston North 4442, New Zealand; rachel.anderson@agresearch.co.nz (R.C.A.); nicole.roy@agresearch.co.nz (N.C.R.); 2 Riddet Institute, Massey University, Palmerston North 4442, New Zealand; 3 High-Value Nutrition National Science Challenge, Auckland 1142, New Zealand

**Keywords:** intestinal barrier maturation, intestinal microbiota, obligate anaerobic bacteria, tight junctions

## Abstract

Appropriate intestinal barrier maturation during infancy largely depends on colonization with commensal bacteria. *Faecalibacterium prausnitzii* is an abundant obligate anaerobe that colonizes during weaning and is thought to maintain colonic health throughout life. We previously showed that *F. prausnitzii* induced Toll-like receptor 2 (TLR2) activation, which is linked to enhanced tight junction formation. Therefore, we hypothesized that *F. prausnitzii* enhances barrier integrity, an important factor in appropriate intestinal barrier maturation. In order to test metabolically active bacteria, we used a novel apical anaerobic co-culture system that allows the survival of both obligate anaerobic bacteria and oxygen-requiring intestinal epithelial cells (Caco-2). The first aim was to optimize the culture medium to enable growth and active metabolism of *F. prausnitzii* while maintaining the viability and barrier integrity, as measured by trans-epithelial electrical resistance (TEER), of the Caco-2 cells. This was achieved by supplementing the apical cell culture medium with bacterial culture medium. The second aim was to test the effect of *F. prausnitzii* on TEER across Caco-2 cell layers. Live *F. prausnitzii* did not improve TEER, which indicates that its benefits are not via altering tight junction integrity. The optimization of the novel dual-environment co-culturing system performed in this research will enable the investigation of new probiotics originating from indigenous beneficial bacteria.

## 1. Introduction

*Faecalibacterium prausnitzii* is one of the most abundant bacterial species in the colon of healthy human adults [1,2]. This bacterium is thought to be critical for maintaining colonic health because its abundance is reduced in people with gastrointestinal diseases [3,4,5,6,7,8,9,10]. Therefore, increasing the abundance of *F. prausnitzii* in the colonic microbiota has become the target of much research, either by directly delivering the bacterium as a probiotic [11] or by using food ingredients that preferentially stimulate the growth of endogenous *F. prausnitzii* [12]. Despite this, little is known about the role of *F. prausnitzii* in appropriate development of the intestinal barrier during infancy and whether it has the potential to be a probiotic during early life.

Intestinal maturation, including the development of the intestinal barrier integrity and immune function as well as the establishment and stabilization of the microbiota, occurs throughout the first two years of life. Much of this process is regulated by diet (e.g., breast milk versus infant formula), which in turn influences the colonization patterns of the early microbiota and their interactions with the host [13]. *F. prausnitzii* colonizes the large intestine between six and 12 months of life [14,15,16], so it is likely to have an impact on intestinal maturation during weaning.

One key area of intestinal maturation is the education of the immune system by the resident bacteria. *F. prausnitzii* has been associated with anti-inflammatory effects in adult gnotobiotic rodents colonized with *Escherichia coli* [17] or *Bacteroides thetaiotaomicron* [18]. However, *F. prausnitzii* is unable to mono-colonize gnotobiotic rodents [17], which means that targeted in vivo studies are not possible. An alternative is to use in vitro techniques to investigate the specific immune-modulatory effects of *F. prausnitzii* on host cells. Such studies have been limited due to the difficulty of co-culturing obligate anaerobes and human oxygen-requiring cells using conventional culturing systems. Using a novel dual-environment co-culturing system we previously showed that live *F. prausnitzii* induced TLR2 activation in transfected human embryonic kidney cells (HEK293) [19], which has been implicated in maintaining homeostasis between immunity and tolerance in the intestinal epithelium [20].

Another key to appropriate intestinal maturation is development of the barrier integrity, which is crucial not only for nutrient absorption but also to prevent the entry of bacteria and food antigens from the lumen into underlying tissues [21]. *F. prausnitzii* improved barrier integrity in mice with DSS-induced colitis [22]. However, our previous study using Caco-2 cell monolayers as a model of the large intestinal epithelium showed that *F. prausnitzii* did not alter ion permeability, as measured by the trans-epithelial electrical resistance (TEER) assay, and increased small molecule permeability, as measured by the ^3^H-mannitol flux assay, which could be considered detrimental [23]. In the study described above using the dual-environment co-culturing system the viability of *F. prausnitzii* in apical anaerobic conditions was improved compared to when cultured in the presence of oxygen, but the bacterium was not actively growing. The discrepancy between the in vivo and in vitro results may be due to this lack of growth, especially since mammalian cells have been shown to respond differently to the same bacterium depending on its growth phases [24].

Therefore, the specific hypothesis of this research was that actively growing *F. prausnitzii* improves intestinal barrier integrity, as measured by the TEER across Caco-2 cells, indicating that it has potential to be a probiotic to improve intestinal barrier maturation during early life. In order to test the hypothesis the first aim of this study was to optimize the apical medium to suit the requirements of both the bacterium and the intestinal epithelial cells, and in particular to encourage growth and active metabolism of *F. prausnitzii*. The second aim was then to test the effects of three *F. prausnitzii* strains, A2-165, American Type Culture Collection (ATCC) 27768, and HTF-F, on TEER across Caco-2 cells to ensure that our results were not limited to one strain.

## 2. Materials and Methods

### 2.1. F. prausnitzii Culturing Conditions

The three *F. prausnitzii* strains A2-165 (DSM 17677), ATCC 27768, and HTF-F (DSM 26943) were kindly provided by Hermie J. M. Harmsen (Department of Medical Microbiology, University of Groningen, Groningen, The Netherlands). Bacteria were cultured anaerobically in Brain Heart Infusion (BHI) broth containing 3.7% (*w*/*v*) BHI powder (Becton Dickinson, Auckland, New Zealand) supplemented with 0.5% (*w*/*v*) yeast extract, 0.0005% (*w*/*v*) hemin, 0.0005% vitamin K and 0.2% l-cysteine (Sigma-Aldrich, Auckland, New Zealand) in an atmosphere of 10% CO_2_, 10% H_2_ in N_2_ at 37 °C (Concept Plus Anaerobic Workstation, Ruskinn Technology Ltd., Bridgend, UK) as previously described [19].

### 2.2. Caco-2 Cell Culturing Conditions

Caco-2 cells (HTB37) were obtained from the ATCC and used in experiments between passage 25 and 35. Caco-2 cells were maintained in cell culture flasks (Corning, New York, NY, USA) in Medium 199 (M199) cell culture medium supplemented with 10% fetal bovine serum (FBS), 1% non-essential amino acids (NEAA; 100× solution), and 1% penicillin-streptomycin (10,000 units/mL penicillin, and 10 mg/mL streptomycin (Life Technologies, Auckland, New Zealand), referred to as M199 Standard medium (M199 Std) and incubated at 37 °C in 5% CO_2_. Medium was replaced twice a week. For co-culture experiments Caco-2 cells were seeded on semi-permeable polyester membranes (Transwell inserts, 6.5 mm diameter, 0.4 μm pore size, Corning, New York, NY, USA) at a density of 8 × 10^4^ cells per insert and cultured for 16–18 days to attain differentiated cell monolayers (TEER over 400 Ω·cm^2^).

### 2.3. Apical Anaerobic Co-Culture Model

**Figure 1:**
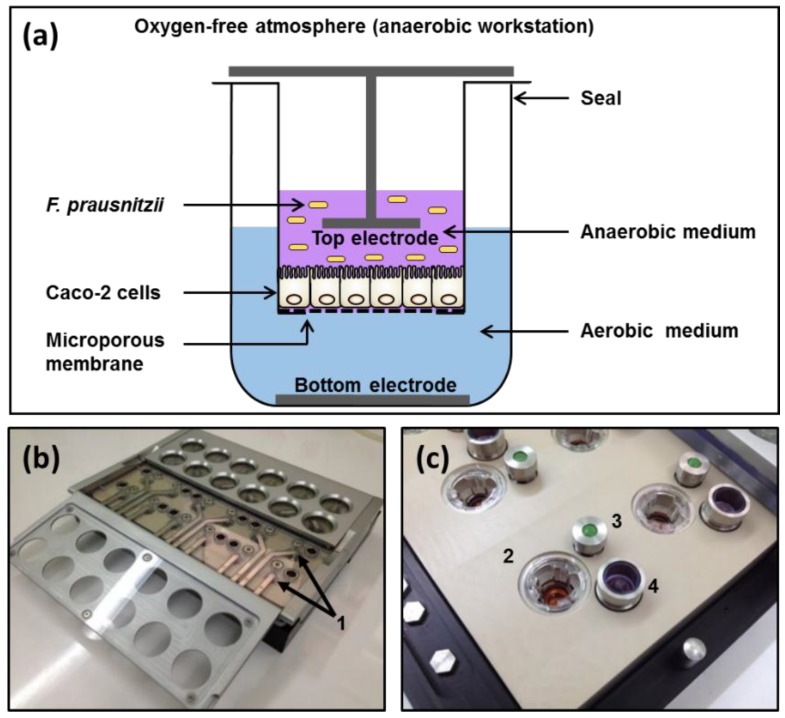
Apical anaerobic co-culture model. (**a**) Schematic diagram of a single well of the apical anaerobic co-culture model used for the co-culture of Caco-2 cell monolayers with anaerobic *F. prausnitzii.* The top and bottom electrodes enable the determination of the effect of *F. prausnitzii* on TEER across the Caco-2 cell monolayers; (**b**,**c**) Photographs of the co-culture chamber including details of the components. 1: Top electrodes; 2: Transwell insert containing Caco-2 cell monolayer; 3: Septum for basal media sampling; 4: One-way pressure relief valve.

The apical anaerobic co-culture model utilized an in-house designed and built dual-environment co-culture chamber inside an anaerobic workstation (Figure 1). The Caco-2 cells received oxygen through the semi-permeable membrane from the oxygenated medium in the basal compartment and the bacterial cells were maintained in the apical anaerobic medium. The chamber was sterilized and consumables and solutions were made anaerobic before each experiment as previously described [19].

### 2.4. TEER Experiment Protocol

Twenty-four hours before the experiments, the apical and basal M199 Std was removed from the Caco-2 cell monolayers grown on Transwell inserts and replaced with M199 supplemented only with 10% FBS and 1% NEAAs, referred to as M199 TEER, in order to remove the antibiotics before co-culturing the cells with bacteria. On the day of the experiment, the basal wells of the co-culture chamber were filled with 3 mL of M199 TEER. The Transwell inserts containing the cell monolayers were carefully inserted into the co-culture chamber using a twisting motion. The co-culture chamber was transferred into the interlock chamber of the anaerobic workstation, purged with nitrogen and after finishing the interlock cycle moved into the anaerobic workstation. The co-culture chamber was connected to the commercially available CellZscope automated TEER monitoring system (CellZscope controller and CellZscope software version 2.2.3; nanoAnalytics, Münster, Germany). The TEER across each Caco-2 cell monolayer was measured twice and the second reading (after 1 h adaptation to the environment) was used as a baseline reading. The apical aerobic medium was removed and replaced with 260 μL of anaerobic medium or treatments. The TEER measurements were resumed and recorded hourly over 12 h. Since the initial TEER for each insert was different, the effect on TEER over time was expressed as the change in TEER compared to the initial TEER for each insert using the following Equation (1):

(1) $$\mathrm{Change}\;\mathrm{in}\;\mathrm{TEER}\;(\%)=\frac{{(\mathrm{TEER}}_{\mathrm{current}}{-\mathrm{TEER}}_{\mathrm{initial}})}{{\mathrm{TEER}}_{\mathrm{initial}}}\times 100.$$

### 2.5. Survival of F. prausnitzii in Anaerobic Cell Culture Media

The three *F. prausnitzii* strains were incubated in M199 TEER medium to monitor their survival. Secondary bacterial cultures in stationary phase were pelleted by centrifugation at 2500× *g* for 6 min (11180/13190 rotor, Sigma 3-18K centrifuge, Osterode am Harz, Germany) and resuspended in anaerobic media inside the anaerobic workstation. Bacterial number was estimated using a Petroff-Hauser counting chamber and solutions diluted to yield a concentration of 2.4 × 10^7^ bacterial cells/mL. This bacterial density was chosen so that when used in the co-culture experiments described below it resulted in a multiplicity of infection (MOI) of 100 bacterial cells per intestinal epithelial cell. Triplicates of the bacterial solutions were incubated at 37 °C and the optical density at a wavelength of 600 nm (OD_600nm_) was recorded (Implen OD600 DiluPhotometer with DC10 DiluCell cuvettes; Total Lab Solutions, Auckland, New Zealand) at 2-h intervals over 24 h.

### 2.6. TEER and Viability of Caco-2 Cells Using a Combination of Cell and Bacterial Culture Media

The effect of combining M199 TEER medium and BHI medium on the TEER and viability of Caco-2 cells was examined using the apical anaerobic co-culture model. Following the measurement of the initial resistance after two hours the apical medium was removed and replaced with 260 μL of anaerobic M199 TEER medium supplemented with increasing concentrations of anaerobic BHI (0%, 25%, 50%, 75%, or 100% BHI). After incubation for 12 h with automated hourly TEER measurements, the inserts were removed and the viability of the Caco-2 cells was determined using the neutral red uptake assay. Neutral red (3-amino-7-dimethylamino-2-methyl-phenazine hydrochloride; Sigma-Aldrich) was dissolved in phosphate-buffered saline (PBS) at 5 mg/mL, filter sterilized (0.22 μm filter), and diluted with M199 Std to a concentration of 50 μg/mL (referred to as neutral red medium). The medium was removed from all the Caco-2 cell monolayers and replenished with 200 μL neutral red medium. After incubation for 2 h at 37 °C in a CO_2_ incubator, the neutral red medium was removed and cell monolayers washed twice with PBS. The dye was extracted from viable cells by adding 200 μL solubilization solution (1% acetic acid–50% ethanol) and incubating at room temperature on a plate shaker at 200 rpm for 7 min. 150 μL of extract was transferred to a 96-well plate and the absorbance of neutral red was determined on a microplate reader at 540 nm (FlexStation 3, Molecular Devices, Sunnyvale, CA, USA). The background absorbance of the 96-well plate was measured at 690 nm and subtracted from the 540 nm measurement. The experiment was completed in five blocks with four replicates per treatment group in each block.

### 2.7. Viability of the F. prausnitzii Strains in the Apical Anaerobic Co-Culture Model Using a Combination of Cell and Bacterial Culture Media

Combinations of anaerobic M199 TEER medium and BHI medium were used for co-culturing Caco-2 cells and the three *F. prausnitzii* strains in the apical anaerobic co-culture model and it was determined if the bacteria were growing in these adapted conditions. The media compositions chosen were anaerobic M199 TEER supplemented with 25% or 50% of anaerobic BHI (referred to as 25% and 50% BHI). This adapted medium was only used for the apical compartment of the co-culture chamber; the basal compartments had aerobic M199 TEER medium. The bacterial suspensions were diluted with 25% and 50% BHI to a concentration of 2.4 × 10^7^ bacterial cells/mL as described previously. The viability of the *F. prausnitzii* strains in the co-culture model was assessed by determining the viable colony forming units (CFU) of the bacteria at 0 h and 12 h after co-culture with the Caco-2 cells in both the cell supernatant and cell lysate. Duplicate ten-fold serial dilutions of the bacterial solutions were made in 96-well plates for each sample. Three 20-μL spots of each dilution were pipetted onto anaerobic BHI agar, allowed to dry, and then incubated anaerobically for 48 h at 37 °C. Spots with between 10 and 100 colonies were counted and the CFU were calculated. This experiment was done in five blocks, with three replicates per treatment group per block.

### 2.8. TEER Assay Using F. prausnitzii in Different Apical Media

To assess the effects of *F. prausnitzii* on TEER, each of the strains (A2-165, ATCC 27768, or HTF-F) was co-cultured with differentiated Caco-2 monolayers in the apical anaerobic co-culture model using 25% or 50% BHI as apical medium. There were eight treatment groups: the two control media (25% and 50% BHI) and the three *F. prausnitzii* strains diluted with the two different media. The TEER was recorded hourly for 12 h. The experiment was done in five blocks, with three replicates per treatment in each block.

### 2.9. TEER Assay Using Live and UV-Killed F. prausnitzii

The effect of both live and UV-killed *F. prausnitzii* on TEER across Caco-2 monolayers in the apical anaerobic co-culture model was determined using 50% BHI as the apical medium. The bacterial suspensions of the three *F. prausnitzii* strains were prepared for the co-culture experiments as follows. A 2-mL aliquot was removed from each bacterial suspension, and transferred to wells of a six-well plate. With the lid removed, the plate was placed on ice and the bacterial suspension treated with a UV lamp (UVP 3UV-38, Bio-Strategy Ltd., Auckland, New Zealand). The bacteria were exposed for 15 min to UVC light. The UV-treated bacteria were plated on anaerobic BHI agar plates and incubated anaerobically for 48 h at 37 °C to confirm that the bacteria were dead. For the TEER experiment there were seven treatment groups: the control medium (50% BHI), the three live *F. prausnitzii* strains and the three UV-killed *F. prausnitzii* strains. The experiment was done in three blocks, with three replicates per treatment per block.

### 2.10. Statistical Analysis

The statistical analyses were performed using SAS (SAS/STAT version 9.3; SAS Institute Inc., Cary, NC, USA). An analysis of repeated measures was conducted to test the effect of the treatment and time and their interaction on the response variables (change in TEER or change in OD_600nm_). The most appropriate covariance structure of the mixed model for each response variable was selected after fitting the models by restricted maximum likelihood method and comparing them using the log-likelihood ratio test. When an interaction was not significant it was removed from the model. An analysis of variance (ANOVA) was also conducted to test the effect of the BHI concentration on the viability of Caco-2 cells in apical anaerobic conditions. When the F-value of the analyses were significant (*p* < 0.05), the means were compared using Tukey tests. A two independent samples *t*-test procedure was performed to compare the viability of the three *F. prausnitzii* strains before and after the co-culture with Caco-2 cells in the apical anaerobic co-culture model. Additionally, a paired *t*-test was conducted to compare the viability of the three *F. prausnitzii* strains before and after the incubation in 50% BHI. For all the analyses the model assumptions (e.g., normal distribution and the homogeneity of variance) was evaluated using the Output Delivery System (ODS) graphics in SAS. When the response variable did not fulfil these assumptions a log_10_ transformation was performed to reach these assumptions.

## 3. Results and Discussion

### 3.1. F. prausnitzii Did Not Grow in an Anaerobic Cell Culture Medium

**Figure 2:**
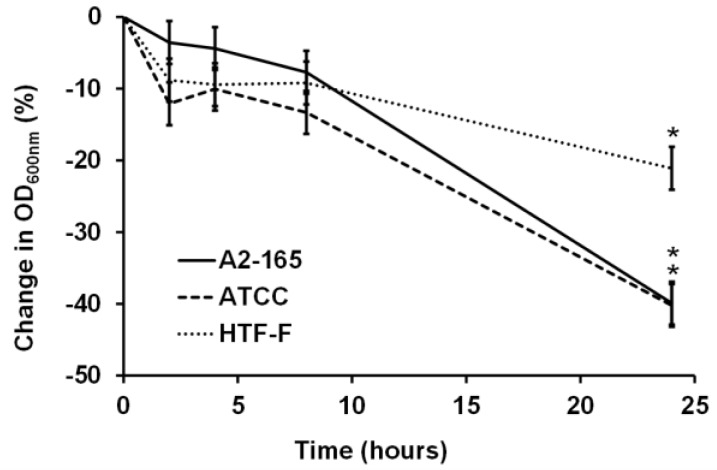
Normalized change in OD_600nm_ (%) of the three *F. prausnitzii* strains in an anaerobic cell culture medium. The three *F. prausnitzii* strains (A2-165, ATCC 27768, and HTF-F) in stationary phase were resuspended in anaerobic M199 TEER, incubated at 37 °C, and the OD_600nm_ was measured over 24 h. The graph shows the mean values (±SEM; *n* = 3) after normalizing by the OD_600nm_ at time 0 h for each of the three *F. prausnitzii* strains over 24 h. * Change in OD_600nm_ different compared to previous time point for the same strain (*p* < 0.05).

For bacterial–mammalian cell co-culture experiments, it is common to harvest bacterial cells by centrifugation and then resuspend the bacteria in mammalian cell culture media [23,25,26]. Studies have shown that there was no difference in the viability for strains of *Escherichia coli*, *Salmonella typhimurium*, and *Lactobacillus fructosus* in mammalian and bacterial culture medium [25]. In contrast to this, our results showed that the three *F. prausnitzii* strains did not grow in the anaerobic cell culture medium over the period of 24 h of incubation at 37 °C, as shown in the change in the OD_600nm_ graph (Figure 2). There were no significant differences in the change of the OD_600nm_ until 8 h of incubation for each of the three strains. However, after 24 h of incubation, all three strains showed a decrease in the OD_600nm_ compared to the previous time points (0 to 8 h; *p* < 0.05). Therefore, it concluded it was necessary to supplement the cell culture medium to stimulate the growth and metabolic activity of *F. prausnitzii.*

When considering which supplements should be added to the medium first the nutrient composition of M199 TEER was investigated. Iron and vitamin K_1_ were identified as nutrients that may be lacking in this medium. Bacterial culture media often contain hemin as the iron source [1,23,27], whereas ferric nitrate is the only iron source in the M199 cell culture medium. This may not be optimum for *F. prausnitzii* since bacteria have developed heme acquisition systems to obtain iron from host heme-sequestering proteins [28]. Similarly, bacterial culture media often contain vitamin K_1_ [27,29], which bacteria convert into the vitamin K_2_ required for electron transport processes during anaerobic respiration [30]; whereas M199 only contains vitamin K_3_ (menadione), a synthetic type of vitamin K, which is a provitamin that requires conversion to menaquinone-4 in order to be active [23,31]. The bioavailability of vitamin K_3_ may therefore be lower for *F. prausnitzii* compared to other vitamin K sources.

Further investigation in the literature indicated that *F. prausnitzii* likely requires a complex medium to grow. A recent study used a combined approach of computational modeling, in vitro experiments, metabolomic analysis and genomic analysis to identify the metabolic capabilities of *F. prausnitzii* A2-165 and to develop a chemically defined medium, CDM1, for this bacterium [32]. However, CDM1 did not enable growth of *F. prausnitzii* A2-165, and even when enriched with additional vitamins, amino acids, and bases, *F. prausnitzii* A2-165 growth was still less than on the bacterial medium YCFAG [32]. Therefore, it was decided to supplement cell culture medium with a complex bacterial culture medium.

### 3.2. Bacterial Medium Did Not Affect Caco-2 Viability, but Reduced TEER at High Concentrations

**Figure 3:**
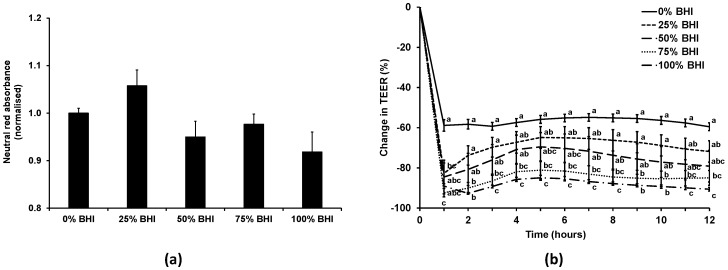
Effect of combining cell and bacterial culture medium on the viability and TEER of Caco-2 cells in the apical anaerobic co-culture model. The apical compartment of Caco-2 cell monolayers were exposed to non-supplemented anaerobic M199 TEER medium (0% BHI), or M199 TEER medium supplemented with increasing concentrations of anaerobic BHI medium (25%, 50%, 75%, or 100% BHI). (**a**) Viability (mean ± SEM; *n* = 20) of Caco-2 cells after 12 h incubation. Neutral red absorbance was normalized by adjusting the 0% BHI exposed cells to 1. Viability of the Caco-2 cells was unchanged as a consequence of differing culture medium composition (*p* = 0.07). (**b**) Mean (±SEM; *n* = 20) change in TEER as a percentage of initial TEER across Caco-2 cell monolayers over 12 h for each medium. There was a significant interaction between the time and the BHI concentration on the change in TEER (*p* < 0.01). Treatments that do not share the same letters are significantly different (*p* < 0.05).

As our previous experience indicated that bacterial medium can be detrimental to mammalian cells, initially the effect of the bacterial medium on the Caco-2 cell monolayers was investigated. The viability of the Caco-2 cells treated with anaerobic M199 TEER mixed with anaerobic BHI in different ratios (0%, 25%, 50%, 75%, or 100% BHI) in apical anaerobic conditions (co-culture chamber inside the anaerobic workstation) over 12 h was determined using the neutral red viability assay (Figure 3a). There was no significant difference (*p* = 0.07) between the viability of Caco-2 cell monolayers treated with 0%, 25%, 50%, 75%, or 100% BHI in the apical anaerobic co-culture model.

The effect of the different M199 TEER-BHI media combinations on TEER across Caco-2 monolayers in apical anaerobic conditions was tested over 12 h (Figure 3b). There was a significant interaction between the time and the BHI concentration on the change in TEER (*p* < 0.01). When treated with 25% BHI, the normalized TEER across Caco-2 cell monolayers was significantly lower at one hour after adding the treatments compared to 0% BHI, however after that time point onwards there was no difference between 0% and 25% BHI. Caco-2 cell monolayers treated with 50% BHI showed no difference in TEER to Caco-2 cells treated with 0% BHI across all time points. In contrast, when 75% or 100% BHI were added to the apical side of Caco-2 cell monolayers, the normalized TEER values were significantly lower compared to cells treated with 0% BHI across all time points (*p* < 0.05).

The TEER of the Caco-2 cells treated with 50% BHI reached a plateau of approximately 300 Ω·cm^2^ following the initial drop in TEER, whereas those with cell culture medium had TEER values that plateaued at approximately 600 Ω·cm^2^. The Caco-2 cell monolayers treated with this combination of bacterial and cell culture medium had TEER values that were more comparable to those reported for human colon tissues (100 to 300 Ω·cm^2^) [33,34].

### 3.3. Two F. prausnitzii Strains Grew in 50% Bacterial Medium When Co-Cultured with Caco-2 Cells

**Figure 4:**
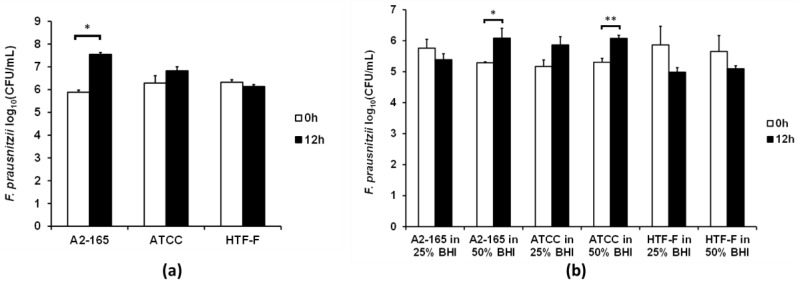
Viability of the three *F. prausnitzii* strains in a cell culture medium supplemented with bacterial culture medium. (**a**) In Hungate culture tubes. The three *F. prausnitzii* strains in stationary phase were resuspended in anaerobic M199 TEER: BHI (1:1) (referred to as 50% BHI) and incubated anaerobically at 37 °C. The graph shows the mean (±SEM; *n* = 3) log_10_(CFU/mL) of the three *F. prausnitzii* strains before and after 12 h of incubation in 50% BHI. * Mean log_10_(CFU/mL) differ between 0 and 12 h at *p* < 0.05. (**b**) In the apical anaerobic co-culture model with Caco-2 cells. The three *F. prausnitzii* strains were co-cultured with Caco-2 cells using two different media on the apical side of the Caco-2 cell monolayer (25% and 50% BHI). The graph shows the mean (±SEM; *n* = 4 and 8 for time 0 and 12 h, respectively) log_10_(CFU/mL) of the bacteria at 0 and 12 h of incubation with Caco-2 cells in the co-culture model. Mean log_10_(CFU/mL) differ between 0 and 12 h at * *p* < 0.05 and ** *p* < 0.01.

Based on the results above, the viability of the *F. prausnitzii* strains was tested using the anaerobic M199 TEER: BHI ratio (1:1), which was referred to as 50% BHI. The CFUs of the three *F. prausnitzii* strains were determined before and after their incubation in 50% BHI over 12 h (Figure 4a). The number of CFU of *F. prausnitzii* A2-165 increased by 1.7 log (*p* < 0.001), while the other two strains showed no significant differences between the CFU before and after 12 h of incubation in 50% BHI.

It was proposed that the *F. prausnitzii* strains may grow better in the presence of intestinal epithelial cells. Therefore the three strains were co-cultured with differentiated Caco-2 cell monolayers in the apical anaerobic co-culture model using M199 TEER supplemented with 25% or 50% BHI medium on the apical side of the cell monolayers. These medium compositions were chosen as they did not compromise the TEER and viability of the Caco-2 cell monolayers. The viability of the bacterial strains was determined by comparing the CFU before and after 12 h of co-culture with Caco-2 cells. No bacteria were able to be cultured from the Caco-2 cell lysate, therefore indicating that none of the three strains adhered to the Caco-2 cells. When 25% BHI was used at the apical side of the Caco-2 cell monolayer none of the three *F. prausnitzii* strains had an increase in CFU (Figure 4b). The number of CFU of both *F. prausnitzii* A2-165 and *F. prausnitzii* ATCC 27768 increased by 0.8 log after the 12 h of incubation (*p* < 0.05) when 50% BHI was used as the apical culture medium. However, there was no significant difference in CFU of *F. prausnitzii* HTF-F at 0 and 12 h of co-culture with Caco-2 cells when 50% BHI was used as the apical culture medium.

In agreement with published results using a simple dual-environment co-culture model [35], the presence of the Caco-2 cells improved the growth of the *F. prausnitzii* strains. This may be due to the presence of mucins. Though Caco-2 cells do not express mucin-2, the predominant mucin in the gastrointestinal tract, they express mucins 3 and 5A/C [36,37]. Furthermore, *F. prausnitzii* may benefit from the oxygen gradient close to the Caco-2 cell monolayer. *F. prausnitzii* uses an extracellular electron shuttle of flavins and thiols to transfer electrons to oxygen [38]. Small amounts of oxygen may diffuse from the aerobic basal compartment of the apical anaerobic co-culture model through the Caco-2 cell monolayer to the apical side. The *F. prausnitzii* strains may be able to use riboflavin (vitamin B_2_), one component of M199, for its extracellular electron transfer, which may benefit growth at this oxic–anoxic interphase [39].

### 3.4. Live F. prausnitzii Did Not Alter TEER across Caco-2 Cells

**Figure 5:**
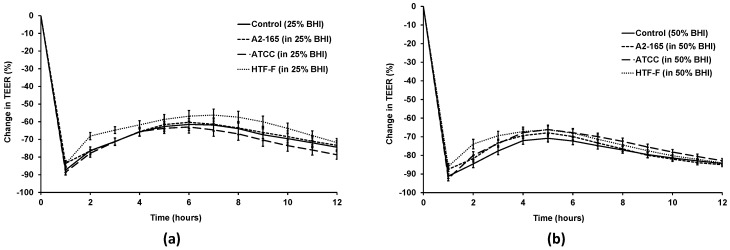
Change in TEER across Caco-2 monolayers co-cultured with live *F. prausnitzii* using 25% and 50% BHI as apical medium. Differentiated Caco-2 monolayers were co-cultured with the three *F. prausnitzii* strains for 12 h in the apical anaerobic co-culture model using 25% or 50% BHI as apical medium. The interaction between the treatment and time was significant for the change in TEER (*p* < 0.001). The graphs show the mean (±SEM; *n* = 12) change in TEER across Caco-2 monolayers when using (**a**) 25% BHI and (**b**) 50% BHI as apical medium. No differences were determined between the two control media and the three *F. prausnitzii* strains in the respective medium at any time point (*p* > 0.05).

To determine whether live *F. prausnitzii* was able to improve TEER, differentiated Caco-2 monolayers were co-cultured with the three *F. prausnitzii* strains (A2-165, ATCC 27768, or HTF-F) in the apical anaerobic co-culture model using 25% or 50% BHI as apical medium. These apical media were chosen as one of them enabled growth of *F. prausnitzii* A2-165 and ATCC 27768 in co-culture with Caco-2 cells (50% BHI), whereas the other medium did not enable growth of any of the three strains (25% BHI) in the previous experiment. It could therefore be determined if change in TEER over time across Caco-2 monolayers differed when co-cultured with growing or non-growing bacteria. The interaction between the bacterial treatment and time was significant for both media (Figure 5; *p* < 0.001). However, there were no differences between the TEER of Caco-2 cells treated with either the 25% or 50% BHI medium or the three *F. prausnitzii* strains in the respective medium for each time point (*p* > 0.05).

Based on these results, it is likely that the maintenance of colonic health by *F. prausnitzii* is not mediated through enhancement of epithelial barrier integrity. Instead, the beneficial effects of *F. prausnitzii* may be due to it supporting immune homeostasis, as previously shown [19]. However, it is possible that metabolites secreted by live *F. prausnitzii* may require an increased treatment time than that undertaken here to exert their effects on intestinal barrier integrity. Specific probiotics and commensal bacteria influence intestinal barrier integrity through secreted metabolites [40,41,42], for example butyrate, a short chain fatty acid produced during bacterial fermentation, enhanced the barrier integrity through the regulation of tight junction assembly [43]. However, the barrier enhancing properties of butyrate occurred only after 24 h of incubation and TEER values reached maximum levels between 48 to 72 h [43]. In order to determine the effects of *F. prausnitzii* on TEER over a prolonged incubation time in this model, further validation studies would be necessary to ensure survival of the Caco-2 cells since the initial validation studies were performed for 12 h [23]. In addition, although *F. prausnitzii* is known as one of the major butyrate producers in the colon [1], it is unknown whether it produced butyrate when using 50% BHI as apical medium; therefore, further studies could analyze the composition of the apical medium after the co-culture of *F. prausnitzii* with Caco-2 cells.

### 3.5. UV-Killed F. prausnitzii Decreased TEER

**Figure 6:**
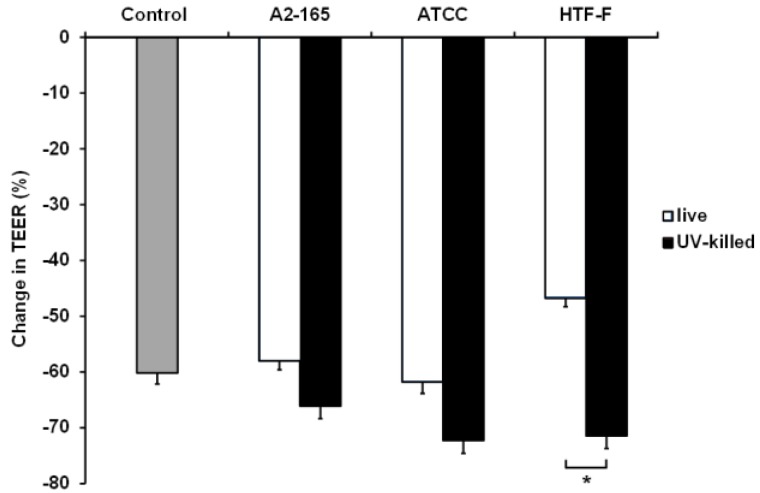
Change in TEER across Caco-2 monolayers co-cultured with live or UV-killed *F. prausnitzii* using 50% BHI as apical medium. Caco-2 monolayers were co-cultured with live or UV-killed *F. prausnitzii* (strains A2-165, ATCC 27768, or HTF-F). The TEER across the Caco-2 monolayers was recorded hourly over 12 h. The interaction between the treatment and time was not significant and so was removed from the statistical model. There was a significant treatment effect on the change in TEER (*p* = 0.002). The graph shows the mean (±SEM; *n* = 9) change in TEER across Caco-2 monolayers co-cultured with live or UV-killed *F. prausnitzii*. * indicates significant difference in TEER (*p* < 0.05).

Caco-2 cells in the apical anaerobic co-culture model were co-cultured with live or UV-killed *F. prausnitzii* (strains A2-165, ATCC 27768, or HTF-F) in 50% BHI for 12 h (Figure 6). The interaction between the treatment and time was not significant, so it was removed from the statistical analysis. There was a significant treatment effect (*p* = 0.002), so between-treatment comparisons were warranted. No differences were observed between the TEER across Caco-2 monolayers exposed to the bacterial treatments and the untreated controls (*p* > 0.05). However, the TEER across Caco-2 monolayers co-cultured with live or UV-killed *F. prausnitzii* HTF-F was significantly different (*p* < 0.05) with higher TEER values recorded for cells treated with live bacteria. It is likely that this detrimental effect of the UV-killed bacteria is due to bacteria surface proteins interacting with the host cells. It is also possible that this negative effect is mitigated by metabolites produced by the live bacterium. Live *F. prausnitzii* may also maintain barrier integrity of Caco-2 monolayers through the activation of innate signaling. For example, commensal induced TLR2 signaling was shown to enhance intestinal barrier function and thereby limit mucosal inflammation [44,45]. We have previously shown that live *F. prausnitzii* induced higher TLR2 activation compared to dead *F. prausnitzii* [19], which may cause the barrier-protecting properties.

## 4. Conclusions

In conclusion, this research resulted in further optimization of the novel dual-environment co-culturing system, which will enable the investigation of new probiotics originating from indigenous beneficial bacteria. Contrary to our hypothesis, actively growing *F. prausnitzii* (strains A2-165, ATCC 27768, or HTF-F) did not improve intestinal barrier integrity, as measured by the TEER of Caco-2 cells. This result indicates that the benefits of *F. prausnitzii* are likely not due to it altering intracellular tight junction integrity.
